# The International American Congress of Medicine and Hygiene, Buenos Ayres, Argentine Republic, May 25, 1910

**Published:** 1910-04

**Authors:** 


For Texas Medical Journal.

### The International American Congress of Medicine and Hygiene, Buenos Ayres, Argentine Republic, May 25, 1910.

The International American Congress of Medicine and Hygiene of 1910 in commemoration of the first centenary of the May revolution of 1810, under the patronage of His Excellency, the President of the Argentine Republic, will be held May 25th in Buenos Ayres, Argentine Republic.

In order to facilitate the contribution of papers and exhibits from the United States, there has been appointed by the President of the Congress, Dr. Eliseo Canton, and the Minister of the Argentine Republic at Washington, a committee of propaganda of which Dr. Charles H. Frazier (Philadelphia, Pa.) is chairman and Dr. Alfred Reginald Allen (Philadelphia, Pa.) is secretary.

The Congress has been divided into nine sections, each section being represented in the United States by its chairman in this Committee of Propaganda as follows:

Section 1. Biological and Fundamental Matters—Dr. W. H. Howell, Chairman, Baltimore, Md.

Section 2. Medicine and Its Clinics—Dr. George Dock, Chairman, New Orleans, La.

Section 3. Surgery and Its Clinics—Dr. John M. T. Finney, Chairman, Baltimore, Md.

Section 4. Public Hygiene—Dr. Alexander C. Abbott, Chairman, Philadelphia, Pa.

Section 5. Pharmacy and Chemistry—Dr. David L. Edsall, Chairman, Philadelphia, Pa.

Section 6. Sanitary Technology—Dr. W. P. Mason, Chairman, Troy, New York.

Section 7. Veterinary Police—Dr. Samuel H. Gilliland, Chairman, Marietta, Pa.

Section 8. Dental Pathology—Dr. George V. I. Brown, Chairman, Milwaukee, Wis.

Section 9. Exhibition of Hygiene—Dr. Alexander C. Abbott, Chairman, Philadelphia, Pa.

It will not be necessary for one contributing a paper or exhibit to the Congress to be present in person. Arrangements will be made to have contributions suitably presented in the absence of the author.

The official languages of the Congress will be Spanish and English.

Members of the following professions are eligible to present papers or exhibits: Medicine, Pharmacy, Chemistry, Dentistry, Veterinary Medicine, Engineering, and Architecture.

Papers may be sent direct to the chairman of the particular section for which they are intended, or to Dr. Alfred Reginald Allen, Secretary, 111 South 21st Street, Philadelphia, Pa.

---

Remember that a syphilitic mucous patch comes quickly, not slowly; it is soft, not indurated; it remains but a short time, not persistently; it is preceded or followed by other mucous patches, and it is apt to be associated with other signs of syphilis.—*American Journal of Surgery.*

---

To differentiate a tender spot from a simulated pain, it will often be observed that pressure on the former causes a decided increase of pulse rate, while in simulation it does not.—*American Journal of Surgery.*

---

Cotton will hold more securely on an applicator if the tip of the latter is dipped in collodion before winding it. The employment of this device will afford a sense of security when applications are made in urethra, bladder or deep sinuses.—*American Journal of Surgery.*

